# A Theoretical Link Between the GH/IGF-1 Axis and Cytokine Family in Children: Current Knowledge and Future Perspectives

**DOI:** 10.3390/children12040495

**Published:** 2025-04-11

**Authors:** Ignazio Cammisa, Donato Rigante, Clelia Cipolla

**Affiliations:** 1 Department of Life Sciences and Public Health, Fondazione Policlinico Universitario A. Gemelli IRCCS, 00168 Rome, Italyclelia.cipolla@policlinicogemelli.it (C.C.); 2 Department of Life Sciences and Public Health, Università Cattolica Sacro Cuore, 20123 Rome, Italy

**Keywords:** growth impairment, growth delay, growth plate, GH/IGF1 axis, cytokines, inflammation, inflammatory bowel disease, juvenile idiopathic arthritis, personalized medicine

## Abstract

Background/Objectives: Growth in childhood and adolescence is influenced by a complex interaction of genetic, environmental, and hormonal factors, with growth hormone (GH) and insulin-like growth factor 1 (IGF-1) playing crucial roles in linear growth and development. However, chronic inflammation, often detected in situations like inflammatory bowel disease and juvenile idiopathic arthritis, can significantly disrupt the GH/IGF-1 axis, causing a relevant growth impairment. Methods: We conducted a retrospective review focusing on the role of cytokines in the GH-IGF-1 axis and growth. Results: Inflammatory cytokines such as tumor necrosis factor (TNF)-α, interleukin (IL)-1β, and IL-6 have been shown to contribute to GH resistance through an array of mechanisms that involve the downregulation of GH receptors and alterations in IGF-1 metabolism. This disruption negatively impacts the growth plate, particularly by impairing chondrocyte proliferation and differentiation, which are essential for proper bone elongation. This review delves into the intricate relationship among growth, chronic inflammation, and GH-IGF-1 axis, emphasizing the contribution of inflammatory cytokines in modulating GH signaling. It also highlights how cytokines can interfere with the molecular pathways that regulate skeletal growth, ultimately leading to growth disturbances in children suffering from chronic inflammatory diseases. Conclusions: The findings underscore the importance of controlling inflammation in affected individuals to mitigate its detrimental effects on growth and ensure that children may reach their growth full potential.

## 1. Introduction

Child growth occurs at varying rates and is conditioned by the tangled networking of genetic and environmental elements [1]. The hormonal regulation plays a key role in this system, with growth hormone (GH), insulin-like growth factor 1 (IGF-1), glucocorticoids (GC), thyroid hormones, and sex hormones serving as central regulators [1,2]. The GH/IGF-1 axis is the primary controller of linear growth, acting through both systemic endocrine effects and local autocrine and paracrine mechanisms [3,4]. GH is synthesized and released by somatotrophs in the anterior pituitary, with its secretion being controlled by growth hormone-releasing hormone (GHRH) (stimulating the release) and somatostatin (inhibiting the release), as well as the GH secretagogue ghrelin [4,5,6]. GH signaling involves the activation of various players, such as signal transducers, activators of transcription [STATs], mitogen-activated protein kinases [MAPKs], insulin receptor substrates, and intracellular calcium [4,5,6]. IGF-1 regulates cell growth, differentiation, and apoptosis: it spreads linked to insulin-like growth factor-binding proteins (IGFBPs), that support its transport, availability, and clearance [5,6]. GH exerts its effects either directly on GH receptors in tissues such as the long bones, spine, liver, muscles, and adipose tissue, or indirectly by promoting IGF-1 production within the liver [6]. Specifically, GH acts on precursor cells in the germinal zone to stimulate chondrocyte differentiation, which leads to increased local IGF-1 formation that induces the enlargement of chondrocyte columns in an autocrine and paracrine way [4,7].

Recent studies have highlighted a connection between chronic inflammation and GH-IGF-1 axis dysregulation, as evidenced by growth impairment in children with protean inflammatory conditions [6,7,8,9]. Chronic inflammation appears to break up the GH/IGF-1 axis through different ways, as deficiencies in GH and/or IGF-1, peripheral resistance to these hormones due to receptor downregulation, disruption in GH/IGF-1 signaling pathways, malfunction of IGFBPs, lower IGF availability, and alterations in gene expression influenced by microRNA (miRNA) [5,10,11].

The aim of this review is to assess the interaction between growth and chronic inflammation with a particular focus on how inflammatory cytokines may influence the GH-IGF-1 axis. It also summarizes the current knowledge of the main mechanisms explored in the medical literature and provides a potential basis to suggest auxological and hormonal evaluations in children with chronic inflammatory diseases as well as new therapeutic approaches.

## 2. The Interplay Between GH-IGF-1 Axis and Cytokines

Cytokines represent a diverse group of messengers that encompass a wide array of molecules, including interleukins (ILs), colony-stimulating factors (CSFs), interferons (IFNs), tumor necrosis factor (TNF), and growth factors [12]. These signalers are secreted by different cell types, such as immune cells, adipocytes, mast cells, fibroblasts, endothelial cells, and keratinocytes, which all contribute in maintaining cellular homeostasis and modulating immune responses. Cytokines can exert their effects on the cells that produce them (autocrine action), on nearby cells (paracrine action), and on cells in distant organs (endocrine action) [7,12,13]. A striking hallmark of cytokines is their pleiotropic nature, which refers to their broad and multifaceted effects [7,12].

The main investigated inflammatory cytokines are TNF-α, IL-1β and IL-6. TNF-α is a glycoprotein primarily secreted by activated monocytes and macrophages, and to a lesser extent by adipocytes, keratinocytes, fibroblasts, neutrophils, endothelial cells, mast cells, and certain lymphocytes [12,13]. It acts through receptors (TNF-R1 and TNF-R2) presented in all nucleated cells, and it both induces the liver to release acute-phase proteins and activates the transcription factor “nuclear factor kappa-light-chain-enhancer of activated B cells” (NF-κB), which subsequently triggers the production of other pro-inflammatory cytokines, including IL-6 and TNF-α itself [12,13]. IL-1β is produced by activated macrophages, monocytes, endothelial cells, and adipocytes and acts linking to the specific membrane receptor IL-R1 type I, which is found on T lymphocytes, keratinocytes, and fibroblasts [12,13]. It stimulates liver to produce acute-phase proteins and local cells to produce IL-2, IL-6, IL-8, TNF-α, and IFN-γ [12,13]. IL-6 is primarily produced by monocytes and macrophages, but is also secreted by T and B lymphocytes, fibroblasts, endothelial cells, and adipocytes. It exerts its effects through the receptor IL-6R, which has tyrosine kinase activity. IL-6 has a central task in controlling many immune responses, initiating and sustaining inflammation by activating a feedback loop involving IL-1 and TNF-α, and contributing to hemogenesis [12,13].

It is important to note that cytokine levels may be influenced by gender and age. Studies conducted on healthy children have shown that cytokine concentrations, particularly TNF-α, were lower in girls compared to boys, as reported by Decker et al. in a study involving 271 children [14]. Wiegering et al. found higher IL-2 expression in males compared to females during the first 6 years of life, while females had a higher number of IL-2-producing cells in the 6-to-12-year aged group and more IFN-γ-producing cells in the 12-to-18-year aged one compared to their male counterparts [15]. Additionally, cytokine levels are age-dependent: a German study reported that IL-6 exhibited a bimodal age-associated pattern, with a peak at 3–4 and 15 years of age, while TNF-α followed a unimodal pattern, peaking at 13–14 years of age. Therefore, the evaluation of these variables should always be considered [16].

**Figure 1:**
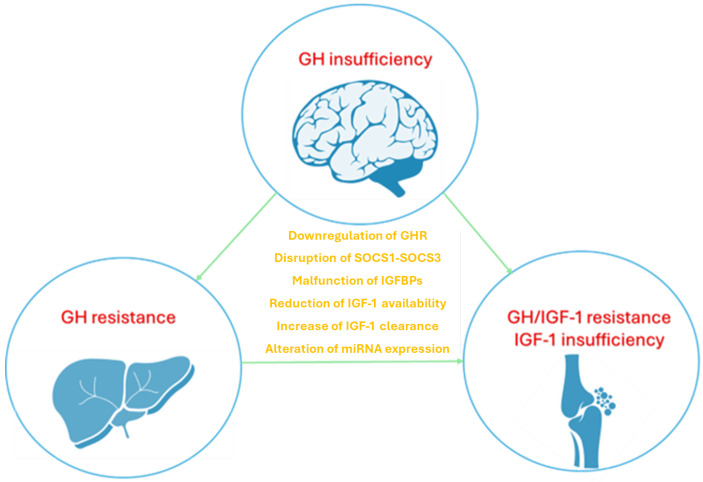
The disruption of GH-IGF-1 axis mediated by inflammatory cytokines involves different mechanisms including altered GH secretion, increased GH resistance in the liver and bones, reduced local IGF-1 availability in bones; the role of cytokines is expressed at different levels disrupting both signaling pathways and gene expression of receptors. Growth hormone (GH), growth hormone receptor (GHR), insulin-like growth factor 1 (IGF-1), suppressor of cytokine signaling (SOCS), insulin-like growth factor-binding proteins (IGFBPs), microRNA (miRNA).

The correlation between inflammatory cytokines and the GH-IGF1 axis has been extensively investigated in inflammatory diseases impacting children’s growth, particularly inflammatory bowel disease (IBD) and juvenile idiopathic arthritis (JIA) [17,18,19]. Several working mechanisms have been suggested to clarify how inflammation may alter GH signaling and its downstream mediator IGF-1, which largely contributes to growth regulation, as reported in Figure 1.

### 2.1. Growth Hormone Resistance: The Dysregulation of Growth Hormone Receptor and Suppressor of Cytokine Signaling

**Figure 2:**
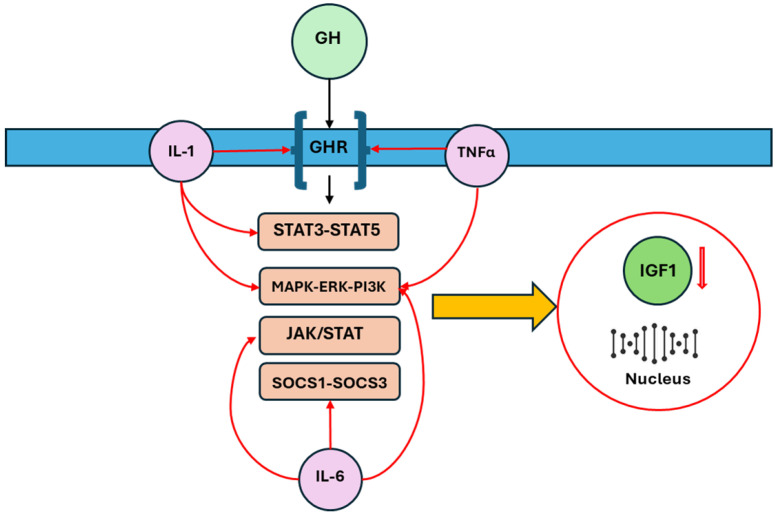
The main intracellular pathways involved in the disruption of GH-IGF-1 axis by inflammatory cytokines are downregulation of the GHR, upregulation of SOCS family, alterations of JAK/STAT, STAT5-STAT3, and MAPK/ERK/PI3K: these changes reflect the production of IGF-1 and its availability. Growth hormone (GH), growth hormone receptor (GHR), insulin-like growth factor 1 (IGF-1), suppressor of cytokine signaling (SOCS), Janus kinase (JAK), signal transducer and activator of transcription (STAT), Mitogen-Activated Protein Kinase (MAPK), extracellular signal-regulated kinase (ERK), phosphatidylinositol 3-kinase (PI3K).

One of the consequences of inflammation is the induction of peripheral resistance to GH, which occurs through two major ways: downregulation of the growth hormone receptor (GHR) and upregulation of members of the suppressor of cytokine signaling (SOCS) family, specifically SOCS1 and SOCS3. These proteins are involved in the negative regulation of GH’s growth-promoting effects, thereby attenuating its action on target tissues [5,20]. Zhao et al. demonstrated that different cytokines have distinct effects on GH signaling [20]. For example, TNF-α and IL-1β were found to inhibit GHR expression with minimal effects on downstream SOCS3, while IL-6 induced SOCS3 expression without affecting GHR expression. In line with these in vitro findings, neutralizing TNF-α and IL-1β in mouse models of inflammation did not significantly modify the expression of SOCS3, which was induced by inflammation, but re-established both GHR and IGF-I expression, both of which had been suppressed by the inflammatory response. On the other hand, neutralizing IL-6 did not affect the GHR expression suppressed by inflammation, but significantly reduced SOCS3 expression and restored IGF-I expression, further suggesting that different cytokines mediate hepatic GH resistance via distinct pathways [20]. Additionally, Denson et al. reported that TNF-α inhibits GHR expression by interfering with the binding of Sp1/Sp3 transactivators to the GHR promoter [21]. Other studies in animal models have shown that GH insensitivity is associated with a decrease in GHR mRNA expression at the receptor level and an increase in SOCS-3 mRNA expression at the post-receptor level, further elucidating the complex regulatory mechanisms involved [22,23]. Additionally, IL-6 may inhibit GH activity by disrupting the JAK/STAT signaling pathway through the expression of SOCS proteins. However, an anomalous expression of STAT5 and STAT3, induced by IL-1β, can interfere with GH signaling. TNF-α, IL-6, and IL-1β also disrupt the MAPK/extracellular signal-regulated kinase (ERK) and phosphoinositide 3-kinase (PI3K) pathways [6,24,25,26,27]. These involved pathways are summarized in Figure 2.

### 2.2. Insulin-like Growth Factor 1 and Insulin-like Growth Factor-Binding Proteins

Beyond their role in inducing hepatic GH resistance, pro-inflammatory cytokines also contribute to reducing IGF-1 activity throughout the body by influencing the metabolism of IGFBPs [5]. IL-6 has been shown to enhance the proteolysis of IGFBP-3 and disrupt the generation of the IGF-I/IGFBP-3/ALS complex. This disruption leads to shortened half-life of IGF-1 and increased clearance of IGF-1, thereby diminishing its biological effect [12]. This phenomenon has been well documented in clinical studies, such as the one conducted by Street et al., who reported that IGF-1 concentration was related to levels of TNF-α, IL-1β, and IL-6 in children with IBD, with IGF-1 levels being elevated during disease relapses [28]. Similarly, Beattie et al. showed that the IGF system, reflected by serum levels of IGF-I and IGFBP-3, exhibited responsiveness to treatment in active Crohn’s disease (CD). During treatment, indeed, median IGF-I levels significantly increased at 0, 2, 8, and 16 weeks recorded as 78 µg/L, 131 µg/L, 119 µg/L, and 133 µg/L, respectively. IGFBP-3 levels also increased at 0, 2, 8, and 16 weeks being 2.4 mg/L, 2.9 mg/L, 3.0 mg/L, and 3.2 mg/L, respectively [29]. Furthermore, De Benedetti et al. found reduced IGFBP-3 levels in patients with JIA, attributed to an increased proteolysis of IGFBP-3, which consequently decreased the formation of the IGF-I complex [30].

### 2.3. The Potential Role of miRNA

In the field of epigenetics, which refers to inherited changes in gene function that do not involve modification within the DNA sequence itself, miRNAs are emerging as an important tool for elucidating different pathophysiological mechanisms [6]. These small, endogenous, non-coding RNAs act as post-transcriptional regulators by linking to specific sites, typically located in the 3′ untranslated regions (3′ UTR) of target messenger RNAs (mRNAs). When there is total complementarity between the miRNA and its target mRNA, this leads to mRNA degradation. In cases of partial complementarity, the miRNA represses translation of the target mRNA [6]. In response to inflammatory signals, miRNAs regulate gene expression, particularly those genes implicated in immune responses, including cytokine production [6]. The miRNAs are involved in the antigen-presenting activity and costimulatory activity of macrophages and dendritic cells, as well as in the development and function of B and T cells [6]. Studies in animal models, particularly in zebrafish and mice, have demonstrated that miRNAs are crucial for regulating hypothalamic function and pituitary development, as well as for the GH-IGF-1 axis [31]. For instance, in zebrafish, the injection of miR-141 and miR-429a mimics into embryos led to a significant reduced expression of GH and GHR. Similarly, in mice with a Dicer1 gene knockout, a reduction in mature miRNAs was observed in the anterior pituitary, which was subsequently followed by growth retardation [31]. A recent study on the rat pituitary gland identified 15 miRNAs that were differentially expressed during the growth process, with 15 miRNAs playing a role in GH signaling. Among these, miR-141-3p was found to negatively regulate GH expression, leading to a decrease in GH1 mRNA levels. In contrast, inhibiting miR-141-3p resulted in a significant increase in GH1 gene expression [31].

Additionally, miRNAs seem to regulate IGF-I levels and its related receptor. Experimental studies in zebrafish showed that miR-141 and miR-429a significantly decreased the expression of IGF-I by directly targeting multiple genes involved in the GH/IGF axis [31]. Moreover, miRNAs regulate the expression and activation of NF-κB, which mediates growth plate chondrogenesis through IGF-1. This process supports longitudinal bone growth by promoting chondrocyte proliferation and inhibiting apoptosis [6,31]. On the other hand, it appears that IGF-1 levels influence miRNA expression: elevated IGF-1 levels are strongly connected with a reduction in the expression of certain miRNAs, while reduced IGF-1 levels correlate with an increase in the expression of these miRNAs [31]. Despite the various findings in animal studies and early in vitro studies on human cells, future research will be necessary to validate these observations in humans, as demonstrated by recent studies. For example, Catellani et al. examined the alterations in circulating miRNAs in individuals with isolated idiopathic GH deficiency (GHD) after the first 3 months of rhGH treatment. They found that miR-199a-5p, miR-335-5p and miR-494-3p were upregulated after 3 months of rhGH, likely reflected both the degree of GH deficiency and the sensitivity to treatment [32].

miRNAs not only target genes encoding proteins that are part of the GH/IGF-1 system but also influence cytokine regulation. In fact, miRNAs have the potential to regulate cytokine expression by directly binding to target sites in the 3′UTRs of mRNAs or through indirect mechanisms involving various pathways, as demonstrated by molecular studies [33,34]. Thus, several studies performed in patients with chronic inflammatory diseases are validating the role of miRNAs as biomarkers [11,35,36,37,38,39,40,41]. Paraskevi et al. compared patients with IBD and healthy controls, finding a higher level of some types of miRNAs, while Kamiya et al. documented altered expression of miRNA in JIA, especially miR-223 [35,36]. However, at present, there are no studies within the field of pediatric chronic inflammatory diseases that correlate specific microRNAs with cytokine levels and specific dysregulations of the GH-IGF1 axis.

### 2.4. Clinical Evidence Linking Cytokines Levels and Growth Impairment

These findings align with earlier research that established a connection between TNF-α and GH levels in children with growth hormone deficiency (GHD). Andiran et al. observed elevated TNF-α levels in children with GHD compared to controls (41.79 ± 25.04 pg/mL versus 8.63 ± 4.48 pg/mL), with a notable reduction after 6 months (13.67 ± 9.95 pg/mL) and 12 months (10.86 ± 6.61 pg/mL) of recombinant human growth hormone (rhGH) treatment [42]. Similarly, Meazza et al. reported comparable reductions in TNF-α levels after 12 months of rhGH therapy, with levels decreasing from 32.0 pg/mL (range 15.6 to 44.0) to 15.6 pg/mL (range 15.6 to 40) [43]. Comparable data were also documented by Serri et al. in studies involving adults with GHD, who had significantly higher baseline levels of TNF-α and IL-6 if compared to controls, with decreased levels after three months of GH therapy [44]. Future studies should determine whether higher concentrations of inflammatory cytokines could predispose to growth impairment, and whether there is a cut-off above which the risk of GH-IGF1 axis suppression is increased.

## 3. Effect of Inflammatory Cytokines on Growth Plate

A normal linear growth in pediatric patients derives from the interaction of various factors, including systemic hormones, cytokines, and growth factors, with the GH-IGF-1 axis having a master position [3]. Longitudinal bone growth occurs through a process known as ‘endochondral’ ossification, where the initial cartilaginous structure is gradually replaced by mineralized bone. This growth takes place at the growth plate, a thin layer located at the ends of long bones between the diaphysis and epiphysis [19]. The growth plate consists of chondrocytes which are found at various stages of differentiation across the resting, proliferative, and hypertrophic zones [19]. GH and IGF-1 are among the most extensively studied regulators of post-natal bone growth, exerting direct effects on the growth plate [7]. A dual effector model of GH/IGF-1 action at the growth plate level has been suggested, where GH directly influences the germinal zone precursors of the growth plate to promote chondrocyte differentiation. This, in turn, stimulates local IGF-1 production, which leads to the enlargement of chondrocytes through autocrine and paracrine effects [45,46].

**Figure 3:**
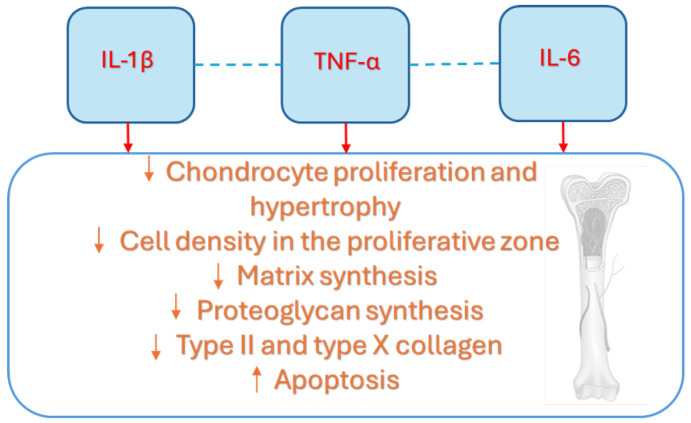
The seminal contribution of cytokines at the growth plate level is largely due to IL-1β, IL-6, and TNF-α, which influence its development, reducing the proliferative stimulation of IGF-1 but also altering its main structure elements, including cell bones, matrix, proteoglycan and collagen. Interleukin-1beta (IL-1β), Interleukin-6 (IL-6), tumor necrosis factor alpha (TNF-α).

Growth impairment in children with chronic inflammatory diseases is linked not only to the systemic effects of cytokines, but also to their local impact on the growth plate via GH/IGF axis, as summarized in Figure 3 [7]. The local effects of cytokines, especially IL-6, IL-1ß, and TNF-α, on bone development have been reported in recent years, with most research focused on their impact on chondrocytes. It has been reported by Martensson et al. that TNF-α could inhibit longitudinal bone growth by direct effects on the growth plate cartilage in a cultured fetal rat metatarsal bone model [47]. Moreover, the authors documented that IL-1ß and TNF-α influenced growth only at higher levels, while their combination had a more powerful inhibitory effect on growth at far lower concentrations [47]. The mechanisms underlying growth suppression included reduced chondrocyte proliferation and hypertrophy, as well as increased apoptosis, assuming that the reduction in proliferative or resting chondrocytes could affect growth outcome in children with inflammatory diseases [47]. Despite the growth promoting effect of administered IGF-I was connected to enhanced proliferation and durability of proliferative chondrocytes, longitudinal growth of metatarsal bone could only be partially restored by IGF-I treatment [47]. Additionally, TNF-α was found to decrease proteoglycan synthesis and mRNA expression of aggrecan, type II collagen, and type X collagen in chondrocyte cell line [26]. A brief duration of exposure of chondrocytes to TNF-α could generally lead to partial recovery, which is not observed in the case of longer periods [26]. IL-1ß also inhibits growth plate chondrocyte differentiation, increases apoptosis, and inhibits cartilage specific proteoglycan synthesis [26,47]. Moreover, it increased DNA synthesis in growth plate chondrocytes, potentially contributing to the excessive endochondral bone growth reported in JIA [48,49,50,51,52]. Recently, it has been found that IL-1β is produced endogenously by growth plate chondrocytes, suggesting that it may contribute to the normal regulation of bone development [50]. However, at higher levels, IL-1β could disrupt bone growth by directly affecting the growth plate [51]. Despite that, in previous studies, IL-6 seemed to have effects on growth plate chondrocytes, recent findings supported the fact that IL-6 could induce growth inhibition through a reduced cell density in the proliferative zone and a decrease in both length and area of the hypertrophic zone [47,48,49,50,51,52,53,54]. Nakajima et al. demonstrated that IL-6 inhibits the early differentiation of chondrocytes by reducing the expression of type II collagen, aggrecan, and type X collagen [55].

Therefore, IL-1β, IL-6, and TNF-α may affect the growth plate by disrupting both the systemic and local effects of the GH-IGF-1 axis, including the IGF-1 intracellular signaling that regulates chondrocyte proliferation and differentiation.

## 4. Growth Outcome in Chronic Inflammatory Diseases

Growth is generally compromised in children with different chronic inflammatory conditions such as IBD, CD, ulcerative colitis (UC), and JIA. Also diseases characterized by spontaneous bouts of inflammation like inherited autoinflammatory diseases starting from an aberrant activation of innate immunity may display additional symptoms like pre- and postnatal growth retardation [56,57,58,59,60,61]. Growth impairment affects up to 56% of children with CD and up to 10% of those with UC, with numerous studies highlighting a final short stature in different JIA patients [60,61,62,63]. It is evident that growth failure for these children results from heterogeneous factors, including the disease itself, malnutrition, reduced physical activity, and treatments like GC. Several trials documented the significance of inflammation, particularly the activity of many cytokines, in this process, as highlighted by the fact that growth impairment typically takes place in the active phase of diseases, while catch-up growth often takes place during periods of remission or when the disease is less active [2]. Increased levels of circulating cytokines have been documented in children with JIA and IBD [64,65,66,67,68,69,70]. During the active phase of JIA, plasma concentrations of TNF-alpha and IL-6 are notably higher, as well as in CD [67,71,72]. At the same time, these patients typically have lower circulating IGF-1 levels, while GH levels remain unchanged, indicating a possible GH resistance [73,74]. Additionally, reduced concentrations of IGFBP-3 may also be present [73,74]. It is important to consider that IGF-1, a useful laboratory marker of the nutritional status, may be lowered in the case of poor nutrition caused by insufficient nutrient intake or malabsorption, which are commonly associated with chronic inflammation [5,75,76]. This correlation can be explained by the effect of pro-inflammatory cytokines on the GH-IGF1 axis. Bozzola et al. suggested that GH resistance in children with JIA could be due to a decrease in the expression of the *GHR* gene, which also correlates with disease activity [73]. Their study found the recovery of both GHR mRNA expression and IGF-1 secretion after two years of JIA-specific treatment [73]. Bechtold et al. also observed reduced pituitary GH secretion in children with JIA, particularly in those receiving systemic GC treatment, while Grote et al. identified a relative resistance to IGF-1, especially in children subjected to long-course treatment with GC [74,75]. Small trials have explored the GH-IGF-1 axis in IBD. Wong et al. reported functional GH deficiency and GH resistance, while Thomas et al. documented lower serum IGF-1 levels in children with active IBD [76,77,78]. Two main therapeutic approaches, rhGH and anti-cytokine therapies, are under study to improve growth outcome in patients with inflammatory diseases.

### 4.1. Recombinant Human GH as Adjuvant Treatment

The use of rhGH on the basis existing between GH-IGF-1 axis and inflammation has been assessed in many trials. One of the first studies was conducted in 1991 by Svantesson et al. in six children with JIA, who had previously received GC for an average of 8.4 years [79]. They were treated with rhGH injections (0.07–0.2 IU/kg/day) for periods lasting from 0.5 to 3 years, documenting after one year of treatment an increase in the mean growth velocity from 2.8 cm/year (range 0.3–5.7) to 6.7 cm/year (range 2.8–12.4), particularly in those with a polyarticular pattern [80]. Touati et al. showed increased levels of IGF-I and IGFBP3 in children with JIA as well as a higher mean growth velocity, which improved from 1.9 to 5.4 cm/year after 1 year of treatment [80]. After discontinuing the treatment, the growth velocity returned to baseline levels within one year, and after two years the height standard deviation score (HSDS) was even lower than before treatment, confirming the beneficial effects of rhGH [80]. Saha et al. in a randomized controlled trial involving 25 prepubertal children who experienced severe growth delay due to JIA treated with rhGH for 6 months, followed by a 6-month placebo phase, showed a significant growth improvement during the rhGH treatment (the median HSDS was +2.09 during GH treatment, and −1.11 during placebo treatment) [81]. Bechtold et al. also documented a height improvement of 1 standard deviation (SD) in children with JIA treated with rhGH, compared to a control group, in whom a reduction of 0.7 SD in height was noted [82]. Disease activity markers were significantly correlated with the average growth velocity: children with milder diseases and lower use of additional medications had better growth outcomes and responded more favorably to rhGH than those with an active disease [82]. However, the effects of rhGH therapy on growth in children with JIA is controversial [83]. For example, Simon et al. demonstrated in children with JIA that during the first 12 months of therapy there was an increased growth velocity, rising from 2.1 to 6.0 cm/year; however, the efficacy diminished over time, and the final HSDS did not show a significant improvement (–4.6 SDS at baseline versus –4.3 SDS at the end of treatment) [84]. Despite evidence that children with JIA who had undergone long-term CS therapy could still experience short stature as adults, even after receiving prompt rhGH treatment, rhGH had significant effects on body composition, increasing lean mass by 33% and preventing further bone loss, with lumbar bone mineral density increasing by 36.6% [85].

Treatment with rhGH was also used in IBD patients. Its initial use in CD was evaluated by McCaffery et al. in 1974, although no improvement in height velocity was reported [86]. Mauras et al. in a perspective study with 10 patients (9 with CD and 1 with indeterminate colitis) who had a history of chronic GC exposure found that growth improved significantly compared to baseline with height velocity increasing from a mean of 3.5 cm/year at baseline to 7.7 cm/year after six months of treatment. Furthermore, serum levels of IGF-1 and IGFBP-3 also improved following this treatment [87]. In a study by Denson et al., 20 pediatric patients with active CD on GC were randomly assigned to receive either rhGH or continue with GC alone. The rhGH group showed an increase in height velocity after one year (height z-score increased from −1.1 (−1.6, −0.6) to −0.4 (−1, 0.2)) [88]. Another study by Wong et al., who randomized 22 children with CD and growth delay to receive either rhGH or a placebo for six months, demonstrated significant increases in median height velocity (HV). Median HV increased from 4.5 at baseline to 10.8 cm/year at 6 months in the rhGH group, whereas in the control group it was 3.8 and 3.5 cm/year, respectively [89]. Currently, only studies examining the short-term effects of rhGH therapy are available. Additional studies with longer follow-up periods are necessary to evaluate the true effectiveness of this treatment on final height.

### 4.2. Anti-Cytokine Therapies and Growth Catch-Up

An effective therapy of chronic inflammatory disorders like IBD and JIA should prevent any growth issues in children. Given the substantial evidence connecting growth impairment to inflammation, controlling inflammation is vital to ensure a physiological growth during childhood and adolescence. Anti-inflammatory medications can positively impact growth by reducing inflammation’s negative effects on the growth plate and GH–IGF axis. Treatment strategies focusing on a direct inhibition of inflammatory pathways through cytokine inhibitors or blockers of their receptors, such as biologics, are increasingly proposed in children with IBD or JIA who do not respond to conventional treatments [90,91,92]. Anti-cytokine treatment, especially anti-TNF drugs, not only suppresses inflammation but also may restore growth velocity. Schmeling et al. observed that children with JIA treated with the TNF-blocker etanercept displayed improvement in growth parameters, IGF-1 and IGFBP3 levels; specifically, growth velocity increased from 3.7 ± 1.2 cm before therapy to 7.6 ± 1.2 cm during the first year of treatment [93]. The mean length-standard-deviation score (SDS) improved from −2.4 ± 1.0 to −1.9 ± 0.9 after one year, and further to −1.1 ± 0.9 after two years (*p* = 0.05), indicating a catch-up growth [93]. Similarly, Tynjälä et al. reported that children with JIA treated with either etanercept or infliximab exhibited an increased growth velocity by +0.45 cm during anti-TNF therapy with a growth rate increasing by +1.8 cm/year [94]. They also showed that inflammatory activity remained a significant predictor of growth velocity, suggesting that the observed improvement in growth velocity could be due to the reduction in inflammation rather than a direct impact of biological agents on skeletal maturation [94]. Furthermore, it has been demonstrated that TNF inhibitors can also improve growth outcomes in patients with hereditary autoinflammatory conditions, in whom all clinical manifestations are consistently mediated by inflammation without any involved infectious or other environmental triggers [95,96].

Tocilizumab, an anti-IL-6 receptor monoclonal antibody, has also been shown to give growth improvement in JIA patients. In fact, Miyamae et al. documented a significant improvement in HVSDS from one year before to one year after baseline (−6.0 ± 4.0 to −2.5 ± 3.9) and decreased GC use that was significantly associated with improved HVSDS in 45 children with systemic JIA [97]. The positive effect of tocilizumab was also assessed by a phase III clinical trial conducted by Bharucha et al., who found an improvement of average height SDS from baseline to year 2 (+0.40), with the mean height velocity being 6.7 ± 2.0 cm per year in polyarticular JIA [98].

In line with these findings, the REACH study—a randomized multicenter open-label trial in pediatric patients with CD subjected to infliximab—revealed a significant increase in height SD scores: this effect was pronounced especially in children with bone age delay of at least one year and in those receiving GC at the start of the trial [99,100]. Other studies, such as those by Malik et al. and Sinitsky et al., have confirmed that infliximab therapy may lead to growth improvement that goes beyond the effects of reduced GC use and pubertal progression of children with CD, underscoring the importance of controlling inflammation to obtain an optimal growth outcome [101,102]. Borrelli et al. documented a significant increase in height Z-scores in 18 children with CD after 6 months of infliximab (from −1.15 ± 0.81 to −0.62 ± 0.99), according to Picher et al. who showed positive catch-up growth following infliximab treatment [103,104].

Table 1 summarizes the main results from the studies cited in the text, regarding the use of rhGH and anti-cytokine therapies in inflammatory diseases.

Furthermore, there are currently no clear guidelines, and studies conducted in an adequate manner are lacking, as several intrinsic factors of chronic inflammatory diseases may alter their outcomes. First of all, GC, which exert potent anti-inflammatory effects and are still widely used in inflammatory diseases, have a broad spectrum of adverse effects, particularly growth retardation and decreased bone mineralization. GC can mimic a GH deficiency state by interacting with the GH/IGF-1 axis. Specifically, GC reduce both GH secretion, due to heightened hypothalamic somatostatin activity and a loss of pulsatile release, and the expression of hepatic GH receptors, leading to a decrease in IGF-1 production [105]. Malnutrition, which is very common in IBD, is associated with resistance to GH, considered as an adaptive response because elevated GH levels may be necessary to maintain euglycemia, whereas decreased levels of IGF-I help to conserve energy during periods of nutritional deprivation [71]. The mechanisms underlying GH resistance may vary depending on the severity and timing of the nutritional deficit. Severe malnutrition can induce GH resistance by reducing the number of GH receptors while less severe nutritional deficiencies are more likely to cause GH insensitivity through post-receptor mechanisms [106]. Another key aspect is the varying level of inflammatory disease activity. Growth is more severely compromised in patients with high disease activity and a greater number of relapses, leading to an increased need for GC, compared to patients with mild disease or in remission. Disease activity levels often correlate with higher cytokine levels, which in turn have a greater impact on the GH-IGF-1 axis and on the nutritional and metabolic status. Thus, all these factors could influence the response to therapies and the growth outcome of these patients, and they should always be considered.

## 5. Puberty in Chronic Inflammatory Diseases

Puberty is often dysregulated with a delayed onset of breast development, testicular enlargement, growth spurts, and menarche in children with inflammatory diseases. It is well established that the GH-IGF-1 axis is strongly interconnected to the hypothalamic-pituitary-somatotropic (HPS) axis, as demonstrated by the expression of GH and IGF-1 receptors also in the reproductive organs and by the evidence that children with GH deficiency and GH insensitivity generally experience delayed puberty and impaired genital development [107,108,109]. GH and IGF-1 exert central effects on hypothalamic gonadotropin-releasing hormone (GnRH) neurons, kisspeptin neurons, and gonadotropin-secreting cells in the pituitary gland. Animal studies showed that the IGF-1 signaling is essential for the maturation of GnRH neurons and synaptogenesis required for the initiation of puberty and that it activates the kisspeptin neurons in the anteroventral periventricular nucleus and promote luteinizing hormone (LH) secretion from pituitary cells [110,111,112]. On the other hand, sex steroids (testosterone and oestradiol) play a role in the neuroendocrine control of GHRH release and in adjusting the body’s sensitivity to GH. This is strongly supported by the evidence that in hypogonadal patients, testosterone replacement therapy boosts GH secretion from the pituitary gland by enhancing the frequency and intensity of GH pulses, while in the deficiency of congenital aromatase, and consequently of estrogen, a reduced secretion of GH and IGF-1 could be detected [113,114]. Moreover, into adulthood testosterone enhances the release of ghrelin, a hormone that stimulates GH secretion, thereby increasing GH bursts [115]. Sex hormones significantly modulate peripheral responsiveness to GH, with this effect being sex dependent. Studies in adults have documented higher IGF-1 levels in males than in females, even in cases of GH deficiency. This discrepancy is primarily attributed to the effect of oestradiol in decreasing liver sensitivity to GH, which reduces IGF-1 release. This occurs through the downregulation of JAK-2 phosphorylation and GH signaling, ultimately leading to an oestradiol-dependent inhibition of IGF-1 secretion from hepatocytes [116,117].

In children with JIA, the most frequent pubertal concern is delayed puberty, along with slow pubertal development, isolated delayed menarche in girls, and a decrease in growth during puberty [118]. For example, Rusconi et al. found that the onset of menarche occurred later in JIA patients compared to both their mothers and normal Italian girls, while Maher et al. documented that 15% of children with JIA experienced delays in puberty, a rate significantly higher than the 1.4% observed in controls [119,120]. Delayed puberty is commonly observed also in adolescents with IBD, especially in females with CD [121,122]. In a study of young patients with CD Ferguson et al. reported that 73% of females who experienced disease onset before puberty had menarche at age 16 or later, with some patients having a delay until their early 20s [123]. Jin et al. reported both primary and secondary amenorrhea in children with IBD, with a higher prevalence observed in patients who have not achieved remission or who have active disease [124]. Brain et al., in a previous review of the medical literature, reported a mean age of puberty onset of 12.6 years for females with IBD, compared to 11.1 years in healthy controls and of 13.2 years for males with IBD compared to 12.4 years in healthy controls [125].

Several mechanisms are thought to potentially contribute to delayed puberty in patients with chronic inflammatory diseases. These include the interaction of cytokines with sex steroid production, either by acting directly on the gonads or by suppressing GnRH secretion, as well as decreased leptin levels, possibly due to inflammation-induced anorexia, leading to a reduction in fat mass [102,126,127]. For example, it has been demonstrated that TNF-α could inhibit steroidogenesis in Leydig cells by suppressing the expression of steroidogenic enzymes at the transcriptional level, while IL-1 could influence the luteinizing hormone-releasing hormone (LHRH) secretion and IL-6 has a negative correlation with reproductive hormones [102,127,128,129]. Despite many mechanisms could be responsible of delayed puberty, such as inhibition of sex steroids either directly by affecting the gonads or by suppressing the secretion of GnRH, the effects of cytokines on GH-IGF-1 axis should be always evaluated.

## 6. Limitations

While our review offers an overview of the interplay between cytokines and the GH-IGF-1 axis in inflammatory diseases in children, it has several limitations. First, the review may not have been exhaustive, as some pediatric studies may not be indexed in PubMed or were not captured through our search. Secondly, the studies on inflammatory diseases are highly heterogeneous in terms of methodology, sample size, disease severity, treatment protocols, and follow-up assessments. One of the main limitations of the studies presented in this review, in addition to the considerable heterogeneity, is the varying impact of rhGH and anti-cytokine therapy, which can be influenced by important factors such as the dosage and duration of GC therapy, the level of inflammatory cytokines, baseline GH-IGF1 values, the malnutrition and metabolic status, and the different levels of disease activity (remission, mild, moderate, severe). Future large-scale studies are needed to further explore the relationship between growth and inflammation, with a special emphasis on novel therapeutic approaches.

## 7. Conclusions

The growth process during childhood and adolescence is influenced by an orchestral combination of genetic and environmental players, with hormonal regulation playing a decisive role. The GH/IGF-1 axis is a fundamental architrave for linear growth, where GH and IGF-1 work together to stimulate bone elongation and development. However, chronic inflammation can disrupt this process, especially in children with inflammatory conditions like IBD and JIA. Many pro-inflammatory cytokines, such as TNF-α, IL-1β, and IL-6, can hinder GH signaling, impair IGF-1 production, and negatively impact growth. Recent research has shown that inflammatory cytokines not only affect systemic GH signaling but also interfere with local mechanisms at the growth plate. This phenomenon results in decreased chondrocyte proliferation, differentiation, and survival, ultimately halting bone growth. Furthermore, cytokines can alter the expression of proteins involved in the GH/IGF-1 axis, such as GHR and IGFBPs, which contribute to GH resistance and IGF-1 dysregulation. The negative impact of chronic inflammation on growth can be alleviated through targeted therapies that aim to specifically reduce cytokine activity. Biologic treatments, especially anti-TNF agents, have shown promise in improving growth outcomes in children with inflammatory diseases by mitigating inflammation and restoring GH-IGF-1 signaling. These therapies have led to notable improvement in growth rate, IGF-1 levels, and overall height of pediatric patients with active diseases. Proper management of chronic inflammatory conditions, focusing on inflammation control, is essential for ensuring normal growth during childhood and adolescence: the development of biologic therapies offers new hope for improved growth outcomes in children with inflammatory diseases, highlighting the importance of addressing both systemic and local effects of inflammation on growth. Future clinical trials are warranted to establish international guidelines for the use of rhGH and anti-cytokine therapies in children with inflammatory diseases, with a special emphasis on their effects on growth outcomes.

## Figures and Tables

**Table 1:** Studies regarding growth outcome in children with inflammatory diseases who received rhGH or anti-cytokine treatments.

Study	Study Design	Sample Size (*N*)	Mean Age (Years)	Patient Population	Treatment	Growth Outcome
Svantesson et al. (1991) [79]	Prospective study	6	11.7–17.1	JIA	rhGH (0.07–0.2 U/kg/day)	The mean pretreatment GR was 2.8 cm (range 0.3 to 5.7), increased to 6.7 cm/year (range 2.8 to 12.4) after 1 year of treatment.
Touati et al. (1998) [80]	Prospective study	14	9.8	JIA	rhGH (1.4 U/kg/week)	Mean GV increased from 1.9 to 5.4 cm/year. After 1 year of rhGH discontinuation, it returned to pretreatment values (1.1 cm/year)
Saha et al. (2004) [81]	Randomized controlled study	25	9	JIA	rhGH (0.1 U/kg/day)	The median SDS for HV was +2.09 (ranging from −7.18 to +9.49) during the 6-month rhGH therapy period and −1.11 (ranging from −10.00 to +1.11) during the placebo phase. The median SDS for height improved from −2.08 to −1.79 during GH treatment and from −2.18 to −2.02 during placebo
Bechtold et al. (2003) [82]	Case–control study	18	10.1	JIA	rhGH (0.20–0.33 mg/kg/week)	The average GV in the rhGH-treated group increased from 2.9 cm/year at the beginning of the study to 7.2 cm/year after 1 year, while it remained stable in the control group at 2.6 cm/year and 3.3 cm/year, respectively. The average height increase in the treated group was 1 SD, while patients in the control group experienced a decrease of 0.7 SD.
Simon et al. (2002) [84]	Retrospective study	24	3.4 ± 2.4	JIA	rhGH (0.46 mg/kg/week)	In the first year of treatment there was an increase in GC from 2.1 to 6.0 cm/year; the effectiveness decreased over time and the final HSDS did not show a significant improvement (–4.6 SDS at baseline compared to –4.3 SDS at the end of treatment)
Mauras et al. (2002) [87]	Prospective study	10	11.9 ± 0.9	CD	rhGH (0.05 mg/kg/day)	Linear GV improved from 3.5 ± 0.4 cm/year with prednisone treatment alone to 7.7 ± 0.9 cm/year after 6 months of rhGH
Denson et al. (2010) [88]	Randomized Controlled Trial	20	7–18	CD	rhGH (0.075 mg/kg/day)	The rhGH group experienced an increase in HV after one year (height z-score improved from −1.1 to −0.4, whereas the control group showed no significant change
Wong et al. (2011) [89]	Randomized Controlled Trial	22	14.7	CD	rhGH (0.067 mg/kg/day)	In the rhGH group, the median HV increased from 4.5 cm/year (range 0.6 to 8.9) at baseline to 10.8 cm/year (range 6.1 to 15.0) at 6 months. The control group showed a median HV of 3.8 cm/year (range 1.4 to 6.7) at baseline, which changed to 3.5 cm/year (range 2.0 to 9.6) at 6 months
Schmeling et al. (2003) [93]	Comparative study	18	NA	JIA	Etanercept	GV improved from 3.7 ± 1.2 cm before therapy to 7.6 ± 1.2 cm in the first year of treatment. The average length-SDS increased from −2.4 ± 1.0 to −1.9 ± 0.9 after one year, and further to −1.1 ± 0.9 after two years
Tynjälä et al. (2006) [94]	Retrospective study	71	9.9	JIA	Etanercept (0.4 mg/kg) Infliximab (3–5 mg/kg)	Patients with previously delayed growth showed an increase in growth rate of +1.8 cm/year while no change was detected in the growth rate for patients who had normal growth prior to treatment.
Miyamae et al. (2014) [97]	Randomized controlled trial	45	8.1 ± 4.2	JIA	Tocilizumab (8 mg/kg)	HVSDS increased from 1 year before to 1 year after baseline (−6.0 ± 4.0 to −2.5 ± 3.9)
Bharucha et al. (2018) [98]	Randomized Controlled Trial	187	11.0 ± 4.0	JIA	Tocilizumab (8–10 mg/kg)	Mean HSDS increased significantly from baseline at –0.6 ± 1.1 to Year 1 at –0.4 ± 1.2 and to Year 2 at –0.2 ± 1.1. The mean HV was 6.7 ± 2.0 cm/year
Hyams et al. (2011) [100]	Randomized Controlled Trial	112	13.3	CD	Infliximab (5–10 mg/kg)	The median height z-score at baseline in the main study was 1.64, which improved by 0.45
Malik et al. (2011) [101]	Retrospective study	28	13.1	CD	Infliximab (5–7 mg/kg)	HV increased from 3.6 cm/year (range 0.4–7.8) to 5.5 cm/year (range 2.1–9.2). In infliximab responders, HV rose from 2 cm/year to 6.4 cm/year, while in non-responders HV remained unchanged
Sinitsky et al. (2010) [102]	Retrospective case series review	16	13	CD	Infliximab (5 mg/kg)	The median height Z score changed from −0.5 (range −2.5 to 0.6) to −0.8 (range −2.8 to 1.4)
Borrelli et al. (2004) [104]	Prospective study	18	13	CD	Infliximab (5 mg/kg)	Height z-score increased from −1.15 ± 0.81 to −0.62 ± 0.99
Picher et al. (2014) [103]	Retrospective study	33	13.5	IBD	Infliximab (5 mg/kg)	Height SDS changed from −0.2 ± 0.2 to 0 ± 0.4. Height SDS in children in remission was notably higher than in those with mild and moderate-to-severe inflammation

Juvenile idiopathic arthritis (JIA), Crohn’s disease (CD), inflammatory bowel disease (IBD), recombinant human growth hormone (rhGH), growth rate (GR), growth velocity (GV), standard deviation score (SDS), height velocity (HV), standard deviation (SD), height standard deviation score (HSDS).

## Data Availability

Not applicable.
